# Supplementary material for: Detection and identification of vacancy defects in antimony selenide
Source: Nat Commun. 2026 Jan 3;17:1413. doi: 10.1038/s41467-025-68153-x (PMC12881623; doi:10.1038/s41467-025-68153-x)
Supplement: Supplementary file 1 — Supplementary Information [file 41467_2025_68153_MOESM1_ESM.pdf]

Supplementary Information for

Detection and identification of vacancy defects in antimony selenide

David J. Keeble[1]*, Theodore D. C. Hobson[2], Julia Wiktor[3], Ethan Berger[3], Marcel Dickmann[4], Mohamed R. M. Elsharkawy[1,5], Werner Egger[4], Jonathan D. Major[2], and Ken Durose[2]

[1]Physics, SUPA, School of Science and Engineering, University of Dundee, Dundee DD1 4HN, United Kingdom

[2]Stephenson Institute for Renewable Energy, Department of Physics, University of Liverpool, Liverpool L69 7ZF, United Kingdom

[3]Department of Physics, Chalmers University of Technology, SE-412 96 Gothenburg, Sweden

[4]Institut für Angewandte Physik und Messtechnik, Universität der Bundeswehr München, D-85579 Neubiberg, Germany

[5]Physics Department, Faculty of Science, Minia University, P.O. Box 61519, Minia, Egypt

Supplementary Note 1: Theoretical calculations

Formation and Binding Energy Calculations. Theoretical calculations were performed to determine the stable configurations and charge states of both monovacancy and divacancy defects in Sb_2Se_3. Structures containing mono- and divacancies were created using the ShakeNBreak algorithm[1]. The resulting structures were relaxed with the cp2k package[2,3] using the PBE0 functional with 25% mixing and an energy cutoff of 400 Ry. The defect formation energy E_f was obtained using,

$$E_{\mathrm{f}} = E_{\mathrm{vac}} - E_0 + \sum_i \mu_i + Q\left(E_{\mathrm{VBM}} + \varepsilon_{\mathrm{F}}\right) + E_{\mathrm{corr}}$$

where E_{vac} and E_0 are the energy of the vacancy and the pristine system, respectively, μ_i are the chemical potentials of the vacancies, Q is the charge state, E_{VBM} is the valance band maximum, ε_{F} is the Fermi energy and E_{corr} is the electrostatic correction. For this latter term, we used the correction scheme introduced by Freysoldt, Neugebauer and Van de Walle[4]. The resulting formation energies for the monovacancy defects are shown in Supplementary Fig. 1 and are found to be excellent agreement with previous calculations[5].

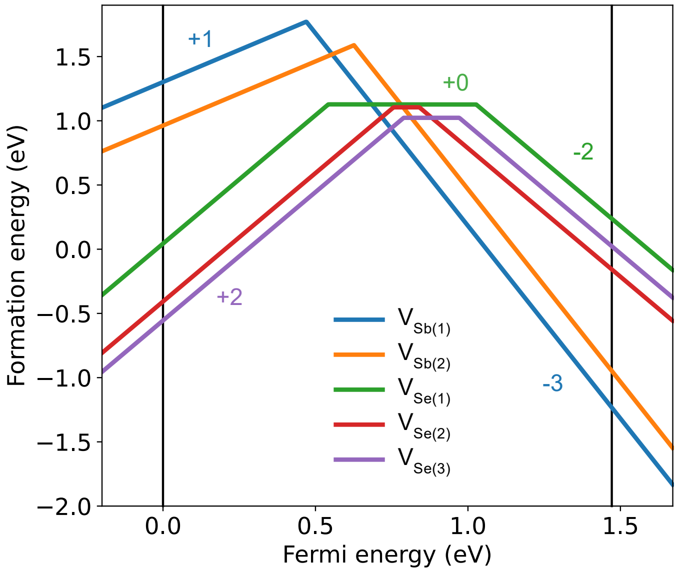

Supplementary Fig. 1. Formation energy of Sb_2Se_3 monovacancies. Colour-coded numbers are used to show the charge states.

The five possible divacancy defects (Supplementary Fig. 2) were investigated and both the binding energy and formation energy of each calculated. The binding energy E_b can be written as the difference in free energy between a divacancy and its two corresponding monovacancies, for example for $V_{Sb(1)}V_{Se(2)}$,

$$E_b = E_f\left[V_{Sb(1)}V_{Se(2)}\right] - E_f\left[V_{Sb(1)}\right] - E_f\left[V_{Se(2)}\right].$$

It should be noted that there are two possible inequivalent configurations for the $V_{Sb(2)}V_{Se(1)}$ divacancy due to the low symmetry the bond lengths from the Sb(2) vacancy site to the two initially equivalent Se(1) sites are different, see Supplementary Fig. 2 (also see Fig. 1), these are denoted as the short- and long-bond configurations in Fig. 2.

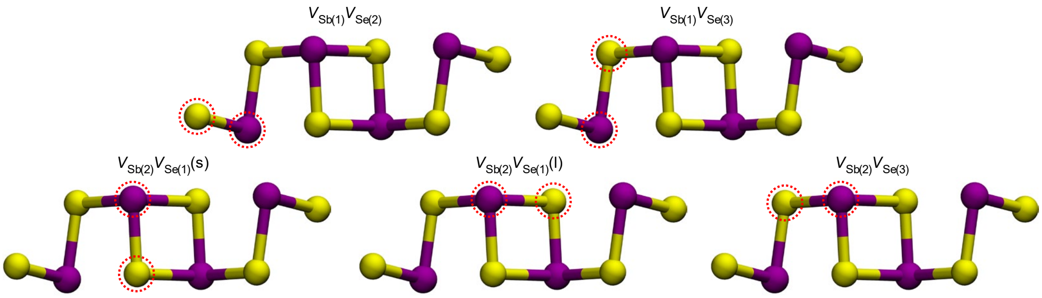

Supplementary Fig. 2. The possible Sb_2Se_3 divacancy configurations. The atoms removed to form the divacancy are denoted by a dotted red circle. For $V_{Sb(2)}V_{Se(1)}$ the bond lengths to the two possible Se(1) nearest neighbours are inequivalent and are denoted as short (*s*) and long (*l*), respectively,

Positron lifetime calculations. Positron annihilation lifetimes were computed within the two-component DFT (TC-DFT) formalism using the ABINIT code[5,6]. The electronic and positronic densities were updated self-consistently in a double loop. During each subloop, one of the two densities was kept constant while the other was converged. To account for the imperfect screening of the positron by the electrons in Sb_2Se_3, we applied the gradient correction proposed by Barbiellini *et. al.*[7,8]. The generalized

gradient approximation (GGA) as parametrized by Perdew, Burke, and Ernzerhof (PBE) was used to describe the electron exchange-correlation interactions[9]. PAW datasets for Sb and Se were taken from the Jollet-Torrent-Holtzwarth (JTH) table and contain 15 and 6 valence electrons, respectively[10]. We used the energy cut-off of 15 Ha. Defect calculations were carried out in $4\times\sqrt{2}\times\sqrt{2}$ supercells containing 160 atoms, based on a unit cell with experimental lattice parameters of 3.96, 11.62, and 11.77 Å[11]. In the case of Se vacancies, where we took initial structures from Ref. 12 containing only 80 atoms, we embedded the geometries in our supercell. The Brillouin zone was sampled only at the Γ point. The defects were relaxed using forces due to both electron and positron densities until convergence below 1 ps was reached. While the TC-DFT calculated positron state lifetime values reported in manuscript were performed using the gradient correction, we also performed tests using the uncorrected Boronski and Nieminen formulation for the electron-positron interaction functionals and enhancement factors, as reported in Supplementary Table 1. We also compare the positron lifetimes calculated for ideal unrelaxed defect structure and the ones fully relaxed according to both forces created by electrons and the positron. The local structures used for the monovacancy calculations were from Wang *et al.*[12] while the divacancy structures were obtain from the calculations described above.

Supplementary Table 1. TC-DFT calculated positron lifetimes (in ps) obtained using ABINIT.

	LDA		GGA	
	Unrelax.	relaxed	Unrelax.	relaxed
Bulk	237		257	
$V_{Sb(1)}^{-3}$	291	301	316	330
$V_{Sb(2)}^{-3}$	292	292	301	314
$V_{Se(1)}^{-2}$				311
$V_{Se(1)}^{0}$				329
$V_{Se(2)}^{-2}$				311
$V_{Se(3)}^{-2}$				302
$V_{Se(3)}^{0}$				332
$\left(V_{Sb(1)}V_{Se(2)}\right)^{-1}$				343
$\left(V_{Sb(1)}V_{Se(2)}\right)^{-3}$				338
$\left(V_{Sb(1)}V_{Se(3)}\right)^{-1}$				344
$\left(V_{Sb(1)}V_{Se(3)}\right)^{-3}$				342
$\left(V_{Sb(2)}V_{Se(1)}\right)_{short}^{-1}$				328
$\left(V_{Sb(2)}V_{Se(1)}\right)_{short}^{-3}$				325
$\left(V_{Sb(2)}V_{Se(1)}\right)_{long}^{-1}$				332
$\left(V_{Sb(2)}V_{Se(1)}\right)_{long}^{-3}$				320
$\left(V_{Sb(2)}V_{Se(3)}\right)^{-1}$				328
$\left(V_{Sb(2)}\ V_{Se(3)}\right)^{-3}$				327

Supplementary Note 2: Further information on Sb_2Se_3 samples

Further information on the Sb_2Se_3 samples measured in the study is given in Supplementary Table 2.

Supplementary Table 2. Sb_2Se_3 sample studied in this work.

Sample name	Doping / conductivity	Sb:Se Stoichiometry (weighed values)	Post-growth treatment	Source material (all Alfa Aesar)	Growth Pressure (mbar)
	Bridgman-grown bulk crystals				
Sb_2Se_3:Sn	Tin (Sn/Sb 0.01 at%); p-type	39.8253: 60.1747	None	99.9999% Sb<br>99.999% Se<br>99.999% Sn	17
Sb_2Se_3:(A)	Undoped; intrinsic	39.9972: 60.0028	None	99.9999% Sb<br>99.999% Se	15
Sb_2Se_3:(B)	Undoped; intrinsic	39.9751: 60.0249	None	99.9999% Sb<br>99.999% Se	17
Sb_2Se_3:Cl(A)	Chlorine; n-type	Commercially supplied	Annealed with 39 mol% Sb/61 mol% Se at 500°C for 168 hrs	99.999% Sb_2Se_3	$4.5x10^{-5}$
Sb_2Se_3:Cl(B)	Chlorine; n-type	Commercially supplied	Annealed with 39 mol% Sb/61 mol% Se at 500°C for 168 hrs	99.999% Sb_2Se_3	$4.5x10^{-5}$
Sb_2Se_3:Cl(C)	Chlorine; n-type	Commercially supplied	Annealed with 55 at% Sb/45% Se at 340°C for 336 hrs	99.999% Sb_2Se_3	15
Sb_2Se_3:Cl,O	Chlorine, oxygen (Sb_2O_3/Sb_2Se_3 0.01 at%); n-type	Commercially supplied	None	99.999% Sb_2Se_3<br>99.999% Sb_2O_3	12
	Thin films				
CSS-as-dep	Chlorine; n-type	Commercially supplied	None	99.999% Sb_2Se_3	
CSS-N_2	Chlorine; n-type	Commercially supplied	Heated under N_2 at 250°C for 20 mins	99.999% Sb_2Se_3	
CSS-air	Chlorine; n-type	Commercially supplied	Heated in air at 250°C for 20 mins	99.999% Sb_2Se_3	

The *n*-type conductivity for the Sb_2Se_3:Cl samples (doping present in commercial source material) was confirmed by the Hall effect as reported in the work Hobson *et al.*[13] and identical processing conditions were used for the Sb_2Se_3:Cl samples applied in this work. Meanwhile, p-type conductivity for Sb_2Se_3:Sn crystals processed identically to those in this work was confirmed by Hall effect measurements reported in the work Hobson *et al.*[14]. Intrinsic samples do not have accompanying Hall effect measurements, as they were highly-resistive, however, in the PhD thesis by T.D.C. Hobson[15] (pg 201 fig. 6.1c) it is demonstrated that no thermally-induced current is observed using the hot-probe technique on identical samples, indicating intrinsic material.

The conductivity of Sb_2Se_3:Cl,O was treated as n-type since a sample Sb_2Se_3:Cl,O (not annealed under Sb) was confirmed n-type via the hot-probe method and a sample Sb_2Se_3:Cl (annealed under identical Sb-rich conditions to the sample in this work) was also confirmed n-type, leading to our reasonable conclusion that the sample in this work, which was subject to both processing methods, would also be n-type. This fact has not been reported previously and so the data on this, and the n-type CSS film samples, can be found Supplementary Figure 3. A heat source was placed on the positive electrode. A negative thermally-induced current corresponds to p-type conductivity while a positive voltage indicates n-type, as confirmed by p- and n-type reference samples. It should noted that because these samples were measured in terms of thermally induced voltage, rather than current, the polarity is reversed with respect to the results of thermally-induced current reported in Ref. 15. The hot-probe results only indicate the conductivity type. Differences in carrier density should not be inferred because sample geometry, thickness and contact size varied between samples.

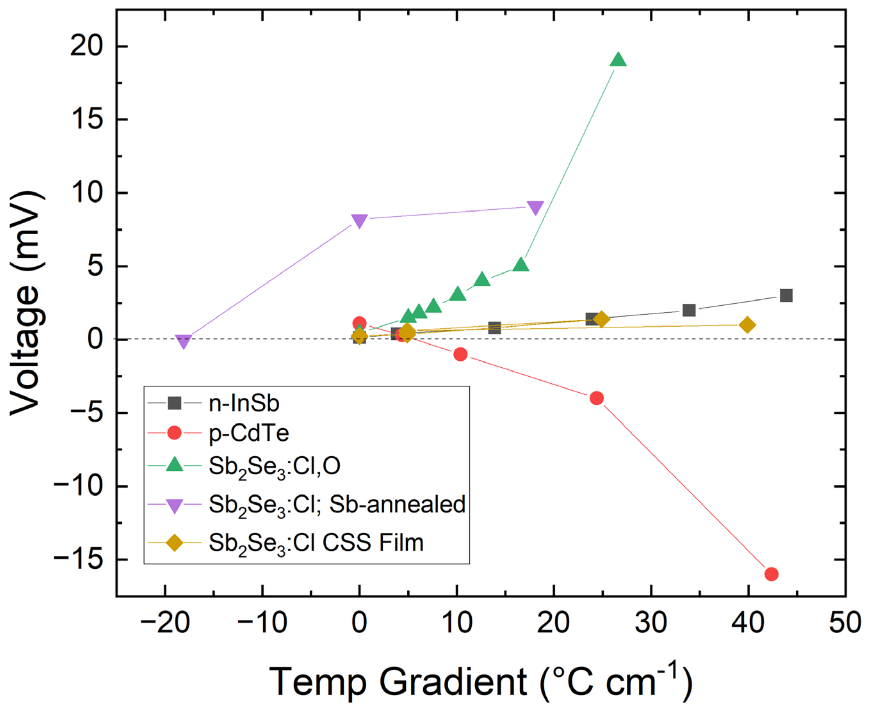

Supplementary Fig. 3. Hot probe data for Sb_2Se_3 samples used in this work.

Supplementary Note 3: Positron lifetime spectrum fits and analysis

The Standard Trapping Model (STM) predicts that the rate of positron trapping, κ_D, is proportional to vacancy concentration, [V], *i.e.* $\kappa_D = \mu_V[V]$, and the constant of proportionality is the defect specific trapping coefficient, μ_V [16]. Assuming the presence of one positron trapping defect the one defect STM (1D-STM) predicts two positron lifetime components, a short first lifetime component with a value less than τ_B the bulk, perfect lattice, lifetime for the material and termed the reduced bulk lifetime component, τ_{RB}, with intensity I_{RB} and a second lifetime component having a value characteristic of the vacancy defect, τ_D, and intensity I_D[16]. The bulk lifetime may be calculated from the experimentally determined lifetime component values,

$$\tau_B = \left(\frac{I_{RB}}{\tau_{RB}} + \frac{I_D}{\tau_D}\right)^{-1} \quad (1).$$

If the STM describes the experimental positron lifetime results the reduced bulk lifetime component intensity I_{RB} reduces as a function of τ_{RB} as described by Eq. 2. Figure 4d shows plot of Eq. 2 assuming a single vacancy defect type with a lifetime of 325 ps and a bulk lifetime of 261 ps as a guide to the eye.

$$I_{\mathrm{RB}} = \frac{\left(\tau_{\mathrm{D}} - \tau_{\mathrm{B}}\right)}{\tau_{\mathrm{B}}\left(\dfrac{\tau_{\mathrm{D}}}{\tau_{\mathrm{RB}}} - 1\right)} \qquad (2).$$

The rate of positron trapping to the defect, κ_D, may be determined using,

$$\kappa_{\mathrm{D}} = \frac{I_{\mathrm{D}}}{I_{\mathrm{RB}}}\left(\frac{1}{\tau_{\mathrm{B}}} - \frac{1}{\tau_{\mathrm{D}}}\right) = I_{\mathrm{D}}\left(\frac{1}{\tau_{\mathrm{RB}}} - \frac{1}{\tau_{\mathrm{D}}}\right) \qquad (3).$$

An approximate vacancy defect concentration, [V], can be estimated from the experimental defect positron rates as $\left[\mathrm{V}\right] = \kappa_{\mathrm{D}}/\mu_{\mathrm{V}}$. The values for defect specific trapping coefficient, μ_{V}, for negatively charged vacancy defects in various semiconductors have been reported to be in the range[16] ~ 1 x 10^{14} – 3 x 10^{16} s^{-1}, given here in units that yield defect concentrations as a number of defects per atom. A more typical value for μ_{V} of ~3 x 10^{15} s^{-1} can be used to estimate vacancy defect concentrations[16]. It is often more useful to give estimated vacancy defect concentrations per unit volume, for this it is necessary to convert defect specific trapping coefficient value given in μ_{V} (s^{-1}) to a value in μ_{V} ($cm^{-3}s^{-1}$) using $\mu_{\mathrm{V}}\left(\mathrm{cm^{3}s^{-1}}\right) = \mu_{\mathrm{V}}\left(\mathrm{s^{-1}}\right)/N_{\mathrm{at}}$ where N_{at} is the number of a particular atom type per unit volume. In consequence, the μ_{V} ($cm^{-3}s^{-1}$) values are material and atom type specific. For Sb_2Se_3 the typical μ_{V} of ~3 x 10^{15} s^{-1} yielded ~1.6 x 10^{-7} $cm^{-3}s^{-1}$ which as an average of the Sb and Se site values. The approximate detect concentrations reported here were calculated from the experiment trapping rates using this value.

The deconvolved lifetime results given in Supplementary Tables 3 and 4 and demonstrate that normally the spectra were dominated by the first two lifetime components and a low

intensity third component normally with a lifetime > 550 ps and attributable to positronium formation. The positronium component is excluded from standard trapping model calculations. Lifetime components with lifetime values less than ~100 ps are difficult to deconvolve reliably preventing the application of the standard trapping model.

The results of the multiexponential decay deconvolutions of the experimental spectra from the p-type and intrinsic samples are given in Supplementary Table 3. Those from the n-type samples are shown in Supplementary Table 4.

Supplementary Table 3| Experimental positron lifetime components for p-type and intrinsic Sb_2Se_3 crystals. Deconvolved component lifetime values, τ, intensities, I, the standard trapping model calculated perfect lattice, bulk, lifetime, τ_B, and positron implantation energy, E.

Sample	Conduct-ivity	E (keV)	τ_1 (ps)	I_1 (%)	τ_2 (ps)	I_2 (%)	τ_3 (ns)	I_3 (%)	$\chi 2$	τ_b (ps)
Sb_2Se_3:Sn	p-type	4	239(4)	62(5)	343(8)	38(5)	1.6	0.2	1.027	270
Sb_2Se_3:Sn	p-type	8	260(1)	96(1)	497(21)	4(1)	5.9	0.1	1.376	260
Sb_2Se_3:Sn	p-type	14	261(1)	96(1)	677(29)	3(1)	4.8	1.1	1.030	261
Sb_2Se_3 (A)	intrinsic	8	182(6)	28(3)	316(4)	71(3)	1.3	0.1	1.153	262
Sb_2Se_3 (A)	intrinsic	14	252(1)	84(1)	467(11)	15(1)	5.4	0.9	1.022	271
Sb_2Se_3 (B)	intrinsic	6	202(3)	43(2)	344(3)	57(2)	1.8	0.1	1.179	264
Sb_2Se_3 (B)	intrinsic	17	258(1)	96(1)	644(9)	4(1)			1.123	258

Supplementary Table 4| Experimental positron lifetime components for n-type single crystal and closed-space sublimation Sb_2Se_3 films. Deconvolved component lifetime values, τ, intensities, I, the standard trapping model calculated perfect lattice, bulk, lifetime, τ_b, and positron implantation energy, E.

Sample	E (keV)	τ_1 (ps)	I_1 (%)	τ_2 (ps)	I_2 (%)	τ_3 (ns)	I_3 (%)	$\chi 2$	τ_b (ps)
Sb_2Se_3:Cl(A)	4	74(3)	13(1)	315(1)	86(1)	1.1(1)	0.7	1.010	
Sb_2Se_3:Cl(A)	8	57(3)	11(1)	306(1)	88(1)	0.8(1)	0.9	1.328	
Sb_2Se_3:Cl(A)	14	55(3)	11(1)	305(1)	88(1)	0.9(1)	0.7	1.067	
Sb_2Se_3:Cl(B)	4	107(4)	10(1)	325(1)	89(1)	1.5(1)	0.9	1.002	270
Sb_2Se_3:Cl(B)	8	62(4)	7(1)	313(1)	92(1)	1.3(1)	0.7	1.181	
Sb_2Se_3:Cl(B)	14	45(4)	7(1)	306(1)	92(1)	0.9(1)	1.2	1.287	
Sb_2Se_3:Cl(C)	4	123(5)	15(1)	311(3)	82(1)	0.65	2.8	1.061	252
Sb_2Se_3:Cl(C)	6	156(6)	20(2)	303(3)	79(2)	0.78	1.7	1.190	255
Sb_2Se_3:Cl(C)	17	258(1)	98(1)	658(30)	2.3(2)	12	0.2	1.192	258
Sb_2Se_3:Cl, O	4	113(3)	16(1)	337(3)	81(1)	0.64	3.5	1.093	254
Sb_2Se_3:Cl, O	6	135(4)	20(1)	329(3)	77(1)	0.67	3.7	1.471	254
Sb_2Se_3:Cl, O	17	188(6)	34(4)	320(7)	73(4)	0.76	3.5	1.031	256
CSS-as-dep	4	110(6)	11(1)	305(3)	86(1)	0.62	3.7	1.080	255
CSS-as-dep	16	192(12)	26(7)	301(7)	67(7)	0.94	3.8	1.172	261
TE-as-dep	4	24(8)	4(1)	354(1)	95(1)	0.62	1.8	1.197	

In this study almost all implanted positrons annihilate directly with electrons, however, in some samples a small fraction form positronium (Ps) prior to annihilation, the bound state of a positron – electron pair. Positronium normally forms in insulators in association with open-volume defects of sufficient size, the process can also occur at surfaces. Positronium forms either in a spin singlet state, para-positronium (p-Ps), or in the triplet state, ortho-positronium

(o-Ps). Para-positronium decays with a lifetime of approximately 125 ps while, in stark contrast, o-Ps has a lifetime of 142 ns in vacuum[16]. When o-Ps forms within a confining within a nanovoid it will annihilate via a pick-off process with a lifetime in the range ~0.55 < $\tau_{\text{o-Ps}}$ < 142 ns, dependent on the size of the open volume. The size of the open volume can be inferred from using a simple quantum mechanical model, the Tao-Eldrup model, but this model must be modified for void sizes greater than approximately 6 nm. The model also breaks down for small open volume sizes which predict o-Ps lifetimes less than 1 ns[17]. In consequence, it is currently not possible to reliable attribute lifetimes in 600 – 800 ps range.

Supplementary References

1. Mosquera-Lois I, Kavanagh SR, Walsh A, Scanlon DO. Identifying the ground state structures of point defects in solids. *NPJ Comput Mater* **9**, 25 (2023).

2. Hutter J, Iannuzzi M, Schiffmann F, VandeVondele J. CP2K: atomistic simulations of condensed matter systems. *WIREs Comput Mol Sci* **4**, 15-25 (2014).

3. Kühne T*, et al.* CP2K: An electronic structure and molecular dynamics software package - Quickstep: Efficient and accurate electronic structure calculations. *J Chem Phys* **152**, 194103 (2020).

4. Freysoldt C, Neugebauer J, Van de Walle CG. Fully Ab Initio Finite-Size Corrections for Charged-Defect Supercell Calculations. *Phys Rev Lett* **102**, 016402 (2009).

5. Wiktor J, Jomard G, Torrent M. Two-Component Density Functional Theory within the Projector Augmented-Wave Approach: Accurate and Self-Consistent Computations of Positron Lifetimes and Momentum Distributions. *Phys Rev B* **92**, 125113 (2015).

6. Gonze X*, et al.* Recent Developments in the ABINIT Software Package. *Comput Phys Commun* **205**, 106-131 (2016).

7. Boronski E, Nieminen RM. Electron-Positron Density-Functional Theory. *Phys Rev B* **34**, 3820-3831 (1986).

8. Barbiellini B, Puska MJ, Korhonen T, Harju A, Torsti T, Nieminen RM. Calculation of Positron States and Annihilation in Solids: A Density-Gradient-Correction Scheme. *Phys Rev B* **53**, 16201-16213 (1996).

9. Perdew JP, Burke K, Ernzerhof M. Generalized gradient approximation made simple. *Phys Rev Lett* 77, 3865-3868 (1996).

10. Jollet F, Torrent M, Holzwarth N. Generation of Projector Augmented-Wave atomic data: A 71 element validated table in the XML format. *Comput Phys Commun* **185**, 1246-1254 (2014).

11. Tideswell NW, Kruse FH, McCullough JD. The crystal structure of antimony selenide, Sb_2Se_3. *Acta Crystallogr* **10**, 99-102 (1957).

12. Wang XW, Kavanagh SR, Scanlon DO, Walsh A. Four-electron negative-U vacancy defects in antimony selenide. *Phys Rev B* **108**, 134102 (2023).

13. Hobson TDC, Phillips LJ, Hutter OS, Durose K, Major JD. Defect properties of Sb_2Se_3 thin film solar cells and bulk crystals. *Appl Phys Lett* **116**, 261101 (2020).

14. Hobson TDC*, et al.* p-type conductivity in Sn-doped Sb_2Se_3. *J Phys Energy* **4**, 045006 (2022).

15. Hobson TDC. Growth and Properties of Bulk CZTSSe and Sb_2Se_3 for Solar Cells.). University of Liverpool (2020) [https://doi.org/10.17638/03104369].

16. Krause-Rehberg R, Leipner HS. *Positron Annihilation in Semiconductors*. Springer-Verlag (1999).

17. Zgardzinska B. The size of smallest subnanometric voids estimated by positron annihilation method. Correction to the Tao-Eldrup model. *Chem Phys Lett* **622**, 20-22 (2015).
